# Epidemiological Survey and Economic Impact of Ruminant Tuberculosis-like Lesions at Slaughterhouses in Two Areas of Northern Algeria (2019–2024): A One Health Assessment

**DOI:** 10.3390/pathogens15050546

**Published:** 2026-05-18

**Authors:** El-Hacene Balla, Omar Besseboua, Nadir Boudjlal Dergal, Sebastian Alexandru Popa, Abdelhanine Ayad

**Affiliations:** 1 Laboratory of Applied Zoology and Animal Ecophysiology, Faculty of Nature and Life Sciences, University of Bejaia, Bejaia 06000, Algeria; elhacene.balla@univ-bejaia.dz; 2 Department of Environment and Biological Sciences, Faculty of Nature and Life Sciences, University of Bejaia, Bejaia 06000, Algeria; 3 Department of Agronomic, Faculty of Nature and Life Sciences, University Mustapha Stambouli, Mascara 29000, Algeria; omar.besseboua29@gmail.com; 4 Laboratory of Biotechnology for Food Security and Energetic, Department of Biotechnology, Faculty of Natural and Life Sciences, University of Oran 1, Ahmed Ben Bella, Oran 31000, Algeria; dergalnadir@gmail.com; 5 Department of Animal Production and Veterinary Public Health, Faculty of Veterinary Medicine, University of Life Sciences “King Mihai I”, Calea Aradului, no. 119, 300645 Timișoara, Romania

**Keywords:** tuberculosis-like lesions, economic impact, ruminants, one health, Algeria

## Abstract

This retrospective study evaluated the prevalence and economic impact of tuberculosis-likelesions (TB) in cattle, sheep, and goats slaughtered at municipal abattoirs in the provinces of Bejaia and Jijel between 2019 and 2024, and examined their ecological association with reported human tuberculosis (TB) cases. The overall prevalence of tuberculosis-like lesions in carcasses, lungs, and livers was 0.08%, 0.85%, and 0.19%, respectively, with cattle showing the highest lesionprevalence. Logistic regression analysis identified species, season, geographic location, and climatic factors as significant predictors of lesion occurrence. Analysis of human tuberculosis records revealed a strong ecological positive correlation (r = 0.81, *p* < 0.05) between bovine pulmonary tuberculosis-like lesions and extra-pulmonary tuberculosis in humans. Over the six-year period, large quantities of condemned carcasses and organs resulted in direct losses of €3.23 million, while reduced carcass weight accounted for indirect losses of almost €11 million.Ruminant tuberculosis-like lesions caused substantial economic losses, totaling €14.16 million over six years, with cattle accounting for 99.8% of the impact. Projected losses could reach €16.7 million by 2030 under comparable surveillance market and control conditions, highlighting the potential ongoing financial burden of the disease. Tuberculosis-like lesions remain relevant in northern Algeria, posing important veterinary, zoonotic, and economic concerns. Enhanced surveillance, laboratory confirmation of suspected lesions, and the strict implementation of control measuresare essential to limit disease transmission and mitigate its impact.

## 1. Introduction

In Algeria, the agriculture sector is central to converging issues of food security and population economics. For many years, the food sector has been the responsibility of the Algerian authorities, who have implemented an investment policy that aims at ensuring a sufficient level of availability in order to support domestic consumption. In recent years, consumption of red meat and its derivatives, which are essential sources of animal protein, has increased in Algeria. This is largely provided by ruminants such as cattle, sheep and goats [1]. However, animal diseases and inadequate herd health management practices pose a significant challenge to efficient herd management and profitable production. Furthermore, Algeria is vulnerable to several transboundary diseases, including tuberculosis, due to its geographical location and its borders with North Africa and the Sahel countries.

Animal tuberculosis (TB) is a chronic, debilitating, and contagious disease. It is characterized by respiratory complications, and also causes enlargement of lymph nodes, emaciation and death [2]. *Mycobacterium bovis*, the bacterium responsible for livestock tuberculosis, belongs to the *Mycobacterium tuberculosis* complex (MTBC) and infects a wide range of domestic animals and also humans. In Algeria, cattle are considered the main hosts of *M. bovis*, which also causes tuberculosis in humans [3]. Animals infected with TB may exhibit non-specific clinical signs, including, but not limited to, the following: nasal discharge, dullness, sneezing, anorexia, weakness, cough, diarrhea, progressive weight loss, pneumonia, enlarged lymph nodes, and dyspnea [4]. Gross lesions compatible with tuberculosis are usually granulomatous and may involve the lungs, lymph nodes, liver, and gastrointestinal tract [5,6]. At the slaughterhouse level, these lesions should be interpreted as tuberculosis-like lesions unless bacteriological or molecular confirmation is available. Mycobacterium pathogens can be transmitted from livestock to humans through the consumption of contaminated meat or milk, or through direct or indirect contact [7,8]. Likewise, the consumption of undercooked meat and organs containing tuberculous lesions, as well as the inhalation of aerosolized particles from infected animals or other individuals, constitute less common transmission pathways for Mycobacterium bovis [9]. It has also been demonstrated that occupational exposure through respiratory transmission or accidental cutaneous inoculation is possible among farmers, animal health workers, slaughterhouse workers, and hunters [10].

Tuberculosis has a substantial impact on various aspects of society and the economy. Numerous recent studies have examined the economic consequences of tuberculosis in livestock [11,12]. Tuberculosis causes significant economic losses, resulting in reduced meat and milk production, as well as the condemnation of carcasses or affected parts deemed unsafe for human consumption. Moreover, this disease has the potential to hinder international trade [13]. Human TB, primarily caused by *Mycobacterium tuberculosis*, has major socioeconomic consequences, including loss of productivity, increased public-health expenditure, and reduced household income [14]. Although the economic burden attributable to animal tuberculosis and *M. bovis* is lower than that of human TB, it remains important for livestock production because of reduced meat and milk yield, carcass and organ condemnation, animal culling, and control-program costs. Therefore, data on the incidence and economic aspects of animal tuberculosis should be evaluated continuously and comprehensively, in order to effectively control and ultimately eradicate the infection in livestock. In Algeria, numerous studies have been conducted on post-mortem tuberculosis-like lesions in animals [15,16,17,18]; however, no investigation has been carried out regarding the economic impact of tuberculosis in slaughtered ruminants. Thus, the objective of this study was to determine the retrospective prevalence and economic losses due to tuberculosis-like lesions in ruminants in different local abattoirs in two regions of northern Algeria. In addition, an exploratory ecological correlation between animal tuberculosis-like lesions and data on human tuberculosis data wasassessed.

## 2. Materials and Methods

Ethical review and approval were waived because this retrospective study used official administrative records collected bythe Provincial Veterinary Inspection and the Department of Public Health of Bejaia and Jijel Provinces, Algeria. No experimental procedures were performed on animals or humans for the purpose of this study.

### 2.1. Study Area

The investigation was conducted in the Provinces of Bejaia (36°45′ N, 5°3′ E) and Jijel (36°49′ N, 5°44′ E), located in northern Algeria, with land areas of 3268 and 2398 square kilometers (km^2^), respectively. The landscape of the study area is characterized by marked variation in relief, with a predominance of mountains that extend towards a Mediterranean coastline, interspersed with narrow coastal plains and fertile valleys.

### 2.2. Slaughterhouse Postmortem Inspection Procedure

Data were obtained from municipal slaughterhouses across the provinces, with all slaughterhouses included in the survey, under the supervision of the Provincial Veterinary Inspection, from January 2019 to December 2024. 

Based on the procedures described by Bensid [19], veterinary inspectors performed routine post-mortem examinations of carcasses, including visual inspection of organs and systematic incision of major lymph nodes to detect tuberculosis-like lesions.

**Figure 1:**
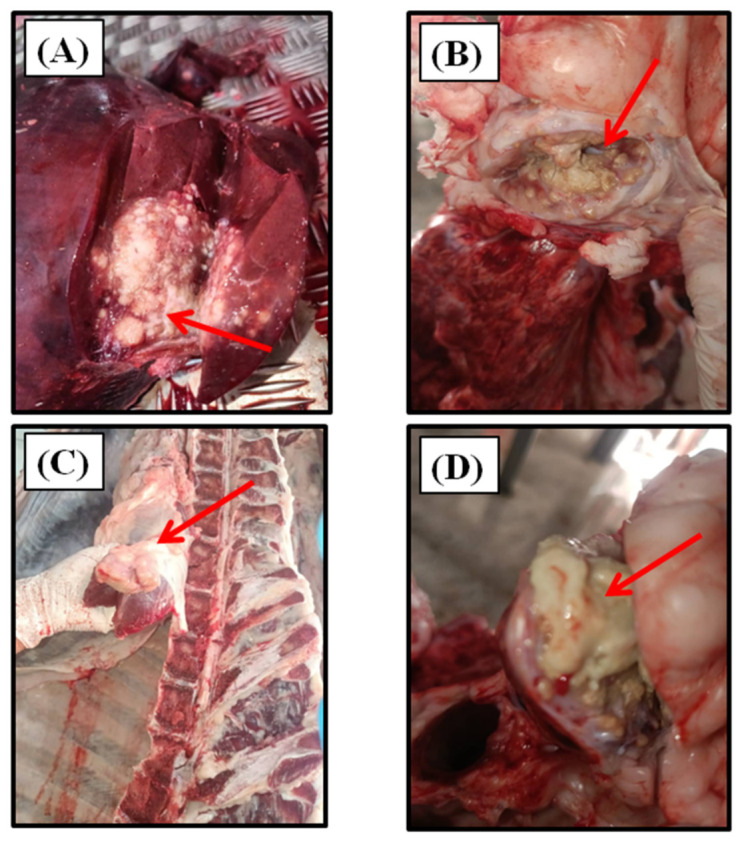
Tuberculosis-like lesions indicated by red arrows on the caseous liver lesions (**A**), lymph nodes of the lungs (**B**), scrotal lymph nodes (**C**), and tracheobronchial lymph nodes (**D**) were observed in abattoirs of Bejaia and Jijel Provinces, Algeria.

A case was defined as any animal presenting lesions compatible with tuberculosis during inspection (e.g., granulomas or caseous lesions in organs or lymph nodes) (Figure 1), leading to either partial or total condemnation. A non-case was defined as an animal with no detectable tuberculosis-like lesions during post-mortem inspection. Condemnation decisions followed standard criteria: total condemnation was applied in cases of generalized or multiple lesions, while partial condemnation was limited to localized and stabilized lesions affecting specific organs or their drainage areas.

Because postmortem inspection alone cannot identify the causative mycobacterial species, all abattoir-based cases in this study were interpreted as tuberculosis-like lesionscompatible with tuberculosis rather than as systematically laboratory-confirmed *M. bovis* infections.

### 2.3. Intradermal Tuberculin (IDT) Test Diagnosis 

The IDT test data were obtained from data compiled annually by the Provincial Veterinary Inspection from January 2019 to December 2024. Tuberculosis screening on farms is carried out by veterinarians affiliated with the veterinary services department. The intradermal tuberculin test was conducted on animals (*n* = 24,177) over six months of age on various dairy farms located in the Bejaia and Jijel regions. A skin test using bovine tuberculin (2000 IU Bovituber^®^ PPD, Synbiotics, Lyon, France) was performed. Purified protein derivative (PPD) is a substance derived from *M. bovis* culture, containing antigens that trigger an immune response in infected animals. For the inoculation of tuberculin, the middle of the neck was shaved, and the thickness was measured with a 0.01 mm graduated caliper; then 0.1 mL of bovine PPD was injected intradermally. The injection sites were examined for swelling and thickness 72 h after inoculation. Each injection site was first visually inspected and palpated for any reactions (swellings), and the skin fold thickness was re-measured. The animal was considered positive when the swelling was 4 mm thicker than the pretest measure.

### 2.4. Data Analysis of Human Tuberculosis 

Tuberculosis data from 2019 to 2024, used to characterize TB epidemiology, were extracted from patient medical records across all hospitals and health facilities included in the survey under the Department of Public Health of Jijel Province. The total population served by hospital facilities in Jijel Province was estimated at 789,667 inhabitants.The extracted data included sex, age, year of diagnosis, and TB classification.

### 2.5. Sample Size Determination

The precision of prevalence estimates was assessed using the standard formula for proportions described by Thrusfield and Christley [20], with a confidence level of 95% and a required absolute precision of 5%.

N = 1.96^2^ × P × (1 − P)/d^2^ (1)

where N = required sample size, P = observed prevalence, d = desired absolute precision at 5%.

### 2.6. Prevalence Determination

The overall prevalence of tuberculosis-like lesions among the three animal species (cattle, sheep, and goats) was calculated using data collected from 2019 to 2024. The number of slaughtered animals infected with TB-like lesions was reported monthly and annually. The annual prevalence (%) was calculated as the number of animals with suspected TB lesions divided by the number of animals examined postmortem. The seasonal prevalence (%) was also determined by calculating the total number of animals with TB-like lesions, recorded across the four seasons (spring, summer, autumn, and winter), divided by the total number of animals slaughtered and examined for each season.

### 2.7. Economic Losses Estimation

Economic losses associated with tuberculosis-like lesions in slaughterhouses in both areas were mathematically estimated as described by Ogurinade and Ogunrinade [21], with slight modification, using the following elements: average organ weight, lesion prevalence, and monthlymarket price per kilogram. The economic analysis was conducted by considering the average monthly selling price of organs and carcasses in the study area. Financial losses were calculated in Algerian Dinar (DZD) and then converted into Euro (€). It is noted that the exchange rate considered varied depending on the year examined.

Direct economic losses due to organ (Equation (2)) and carcass (Equation (3)) condemnation were assessed by considering the prevalence of tuberculosis-like lesions detected at abattoir inspection and the retail market price of average organs and carcasses.

DELo = (MAS × PLr × CLr) + (MAS × PLu × CLu)(2)

where DELo = direct economic losses due to organ condemnation caused by tuberculosis; MAS = mean annual cattle slaughtered at the study area; PLr = percentage of liver condemned; PLu = percentage of lung condemned; CLr = mean cost of a liver; and CLu = mean cost of a lung.

DELc = NC × ACW × ACP(3)

where DELc = direct economic loss due to carcasses condemnation by tuberculosis; NC = number of condemned carcasses; ACW = average carcass weight (kg); ACP = average carcasses price (Euro/kg).

Indirect economic losses were assessed by estimating the reduction in carcass yield of animals. To calculate the indirect economic losses due to BTB, a 10% weight loss due to TB was used as reported by Kwaghe et al. [22]. In this current study, the average weight of cattle, sheep and goats carcasses isestimated at 236, 24 and 17 kg, respectively, in order to obtain the percentage weight of carcass reduction due to TB.

IEL = MAS × PCW × MCM × P(4)

IEL = MAS × (ACW × 0.1) × MCM × P

where IEL = indirect economic losses due to tuberculosis-like lesions; MAS = mean annual ruminants slaughtered in the study area; ACW = average carcass weight; PCW = percentage of carcass weight reduction (ACW × 0.1); MCM = mean cost of 1 kg meat in the study area (Euro); and P = prevalence of tuberculosis-like lesionsin thestudy area.

### 2.8. Projection of Economic Losses

In order to characterize temporal trends in economic losses and generate robust projections for the period 2025–2030, a log-linear regression model was fitted separately for each species (cattle, sheep, and goats). The selection of this approach was made with the intention of capturing proportional changes over time, whilst also stabilizing variance across the short historical time series (2019–2024). This approach is extensively endorsed in econometric forecasting literature for its efficacy in managing non-linear growth trends [23,24]. The model is defined by the following equation:

ln(Loss*_t_*) = a + b × ln(t) (5)

where Loss*_t_* = total annual economic loss (direct + indirect) in Euros (€) for year t; t = time index (t = 1 for 2019, t = 2 for 2020, t = 3 for 2021, t = 4 for 2022, t = 5 for 2023, t = 6 for 2024); a = Intercept parameter representing the baseline logarithmic loss; and b = slope parameter representing the elasticity of losses with respect to time.

The log-linear model implies the following power–law relationship: Loss_t_ = exp(a) × t^b^. In this formulation, b represents the elasticity of economic losses with respect to the time index; therefore, b > 0 indicates an increasing trend, b ≈ 0 indicates approximate stability, and b < 0 indicates a decreasing trend. The model parameters a and b were estimated using Ordinary Least Squares (OLS) on log-transformed annual economic-loss data from the retrospective survey period. Projections for 2025 (t = 7) through 2030 (t = 12) were obtained by extrapolating the fitted model and should be interpreted as exploratory trend-based estimates rather than precise forecasts.

These projections should be interpreted as exploratory scenario-based estimates rather than precise forecasts. The model assumes that surveillance intensity, slaughterhouse inspection procedures, slaughter volumes, market structure, and disease-control efforts remain broadly comparable to those observed during 2019–2024. Because meat prices, exchange rates, and disease prevalence may change over time, the projected values mainly indicate the potential magnitude and direction of the future economic burden in the absence of major control improvements.

### 2.9. Statistical Analysis

All the data were entered, stored, and calculated in Microsoft Excel 2007. The retrospective data were analyzed using the Statistical Package for the Social Sciences (SPSS) version 21.0 (SPSS Inc., Chicago, IL, USA). The data were also presented using descriptive statistics in table and figure form. One-Way Analysis of Variance (ANOVA) and Tukey’s HSD posthoc test were used only as complementary descriptive comparisons across grouped categories (years, months, and seasons). Their interpretation was kept cautious because temporal observations may not be fully independent. The independent-sample t-test was applied for pairwise comparisons when appropriate. Spearman’s rank correlation coefficients were calculated to explore ecological associations between animal tuberculosis-like lesions and reported human extrapulmonary tuberculosis (EPTB) cases; these correlations were not interpreted as evidence of individual-level transmission. Moreover, logistic regression models were additionally performed with infection status as the dependent variable, and species (cattle, sheep, and goats), season (winter, spring, summer, and autumn), years (2019–2024), and areas (Bejaia and Jijel) as independent predictors. In this analysis, a *p*-value less than 0.05 at the 95% confidence level was considered statistically significant.

The probability *P_i_* of tuberculosis-like lesion occurrence in animal *i* was modeled using logistic regression as follows:

Logit(*P_i_*) = ln[*P_i_*/(1 − *P_i_*)] = β0 + β1(Species_i_) + β2(Season_i_) + β3(Region_i_) + Σ(k = 2019 to 2023) βk(Year_k,i_) + β4(Humidity_i_)

where *P_i_* is the probability that animal *i* presents tuberculosis-like lesions; β0 is the intercept corresponding to the reference categories; β1, β2, β3, and β4 represent the effects of species, season, region, and relative humidity, respectively; species, season, region, and year were included as categorical dummy variables; Year_k,i_ represents the year-specific dummy variables, with 2024 used as the reference year; and Σ denotes the inclusion of multiple year-specific dummy variables in the model.

## 3. Results

Table 1 summarizes the number of slaughtered animals examined and the occurrence of tuberculosis-like lesions during the six-year period from January 2019 to December 2024. A total of 367,726 ruminants were slaughtered and inspected during the study period in the provinces of Bejaia and Jijel, including 179,549 cattle (48.8%), 75,634 sheep (20.6%), and 112,543 goats (30.6%). The overall prevalence of tuberculosis-like lesions in carcasses, lungs and livers was 0.08%, 0.85%, and 0.19%, respectively, corresponding to 291, 3125, and 712 recorded cases. The distribution of infection by tuberculosis in slaughtered cattle showedsignificantly higher lesion prevalence than in sheep and goats (*p* ≤ 0.05). The estimated desired absolute precision (d) for carcass, lung, and liver lesion prevalence was 0.0006%, 0.0003%, and 0.0008%, respectively. Among the 24,177 IDT tests carried out on dairy farms, 278 cattle were positive, corresponding to a prevalence of 1.15% and an absolute precision of 0.0015%.

Regarding human tuberculosis, the retrospective study was carried out using observed medical-record data from the Public Health Directorate of Jijel Province, covering the period from 2019 to 2024 (Table 2). These values correspond to recorded cases and were not model-estimated. A total of 1319 tuberculosis cases were recorded, including lymph node, pleural, peritoneal, and other forms (713, 100, 156, and 353 cases, respectively). More tuberculosis patients were female compared to male (387 vs. 247). Depending on age, patients aged 25 to 45 were the most affected, with 286 cases. The number of tuberculosis cases recorded annually was practically similar throughout all years (2019–2024) during this retrospective study. The highest ecological correlation was observed between bovine pulmonary tuberculosis-like lesionsand human EPTB (r = 0.81, *p* < 0.05). A significant inverse correlation was identified between bovine hepatic tuberculosis-like lesions due to tuberculosis and human EPTB (r = −0.81, *p* < 0.05). Other elevated negative correlations were observed but were not statistically significant (*p* = 0.18), including the association between positive IDR results and human EPTB (r = −0.49). These findings should be interpreted as exploratory ecological associations rather than proof of direct zoonotic transmission.

**Figure 2:**
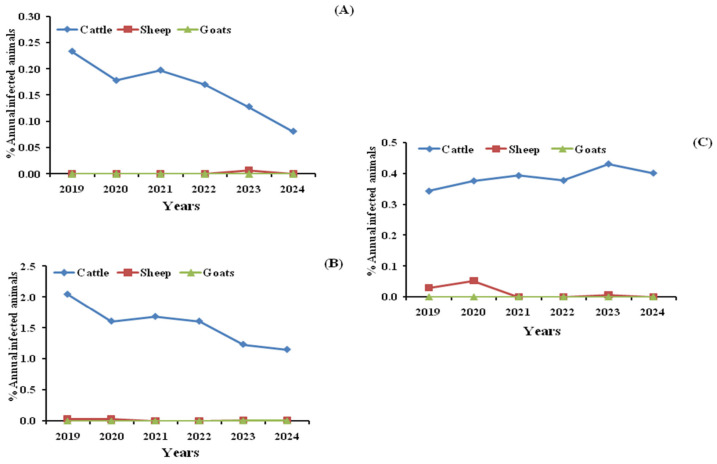
Annual variations in the prevalence of tuberculosis-like lesions in carcasses (**A**), lungs (**B**) and livers (**C**) among slaughtered ruminants during the years 2019-2024 in Bejaia and Jijel Provinces.

Annual trends in the prevalence of tuberculosis-like lesions in carcasses, lungs, and livers during 2019–2024 are shown in Figure 2. In sheep, annual lesion prevalence remained very low and did not differ significantly (*p* > 0.05) in sheep. In goats, no carcass or liver lesions were recorded during the study period;a maximum rate of tuberculosis in the lungsof 0.01% was observed in 2023 and 2024. The maximum rates of bTB were 0.23 and 2.05%, and a minimum of 0.08 and 1.15% in cattle carcass and lung, respectively. As for tuberculosis in cattle liver, the recorded prevalence remained constant during the first four years of the study, and then increased significantly in 2023 and 2024 (*p* ≤ 0.05). These temporal comparisons should be interpreted cautiously because ANOVA and Tukey’s tests were used as complementary grouped comparisons rather than as definitive time-series models.

Multivariate logistic regression analyses were used to determine the strength of the association between the occurrence of tuberculosis and its risk factors (Table 3). Cattle were the most affected species and served as the reference group. The odds of infection were significantly negligible in sheep (OR = 0.03, CI 95%: <0.01–0.06, *p* < 0.001) and goats (OR = 0.02, CI 95%: <0.01–0.05, *p* < 0.001) when compared to cattle. Concerning the seasonality, autumn showed a high risk of infection (OR = 1.18, CI 95%: 0.92–1.5, *p* = 0.058), and summer showed a reduced risk of infection (OR = 0.82, CI 95%: 0.63–1.06, *p* = 0.126) compared to the season reference (winter). Spring was stable in terms of infection risk versus the winter season (OR = 0.95, *p* > 0.05). Using 2024 as the reference, infection odds were lower in 2019 (OR = 0.71), and moderate risk in 2020 (OR = 0.89) and 2023 (OR = 0.85), indicating a rising trend towards the 2024 peak. The province of Jijel presented a significantly lower risk of tuberculosis infection than the province of Bejaia (OR = 0.68, *p* = 0.009), which represents a risk reduction of 32%. Increased rainfall was associated with higher infection odds (OR = 1.06 per 10 mm, *p* = 0.023), while higher temperatures reduced infection risk (OR = 0.96 per 1 °C, *p* = 0.073). Relative humidity showed a non-significant trend. 

Table 4 summarizes the total quantitiesof carcasses and organs condemned because of tuberculosis-like lesions and their corresponding direct and indirect economic losses in slaughtered ruminants from Bejaia and Jijel abattoirs during 2019–2024. Total economic loss was estimated by combining losses from carcass and organ condemnation (direct losses) and losses attributed to reduced carcass weight (direct losses). Over the six-year period, 31,755 kg of carcass, 10,374 kg of lung, and 4061 kg of liver were condemned. Direct losses amounted to €3,233,969, whereas indirect losses due to carcass-weight reduction reached €10,924,250, giving a total estimated economic loss of €14,158,219.

**Figure 3:**
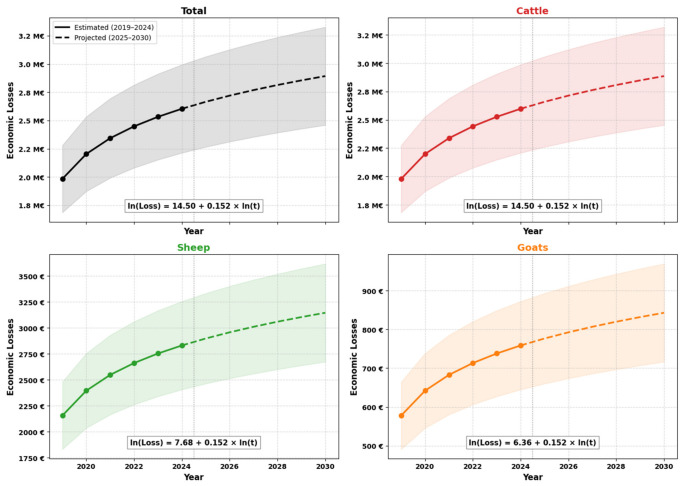
Temporal trends of economic losses due to ruminant tuberculosis between 2025 and 2030 in slaughterhouses of Bejaia and Jijel provinces.

The projection of economic losses related to the condemnation of carcasses due to tuberculosis-like lesions in ruminants slaughtered for the upcoming six-year period (2025–2030) is summarized in Table 5. The log-linear model suggested a consistent upward trend for all species under the assumption that surveillance conditions, slaughter volumes, and control intensity remain broadly comparable to the retrospective period (Figure 3). Cattle represented 99.8% of the total financial impact, and the model yielded a positive slope coefficient (b = 0.152), indicating a gradual increase over time. Specifically, the projected annual economic losses associated with cattle are expected to rise from €2.66 million in 2025 to €2.89 million by 2030. A comparable proportional trend is observed for small ruminants, although their absolute financial contribution remains negligible. Over the 2025–2030 period, the cumulative total economic loss is projected to amount to €16.70 million.

## 4. Discussion

It is imperative for national health authorities to conduct epidemiological surveys, given the pivotal role these investigations play in identifying the sources of diseases such as tuberculosis. Once an outbreak has been identified, the subsequent step involves implementing effective control strategies aimed at preventing the further spread of the disease. The effectiveness of meat inspection in slaughterhouses for the detection of zoonotic infections has long been considered an important tool. Tuberculosis is endemic in many developing and emerging countries, exerting a negative impact on both animal productivity and public health [25]. The zoonotic transmission potential of Mycobacterium bovis from cattle to humans was acknowledged more than a century ago [26]. Globally, statistical data reveal that approximately 10 million people develop TB each year, primarily caused by *M. tuberculosis*, with about 1.2 million deaths [27]; however, these global figures should not be interpreted as representing the specificburden attributable to *M. bovis*. In Algeria, the authorities responsible for animal health have made considerable efforts to eradicate TB in cattle through the implementation of disease control programs. Nevertheless, numerous scientific surveys have reported the persistence of TB on livestock farms, leading to substantial direct and indirect economic losses [18,28,29]. This epidemiological study was conducted over a six-year period (2019–2024) in order to provide a comprehensive description of the incidence of TB in ruminants and the resulting financial losses. The study was based on detailed records from various slaughterhouses in two provinces of northern Algeria.

The present study revealed that cattle were slaughtered more frequently than sheep and goats (48.8%, 20.6%, and 30.6%, respectively). This finding indicates that the high demand for red meat in the studied regions is predominantly focused on beef. It is noteworthy that the proportion of slaughtered ruminant species varied across geographical areas during the study period [18]. The observed discrepancy can be attributed to fluctuations in consumer demand, which in turn are influenced by the prevailing socioeconomic conditions of the country. Furthermore, the timing of various social events, such as weddings and circumcisions, may also contribute to the variability in meat demand. In the present study, post-mortem inspection detected tuberculosis-like lesions mainly in cattle, whereas sheep and goats showed negligible lesion prevalence. This pattern may reflect differences in susceptibility, husbandry, and exposure intensity. The very low prevalence observedin small ruminants may be partly explained by limited cohabitation with cattle and reduced grazing or watering areas, which can lower interspecies transmission risk [30]. Sheep are also generally considered less susceptible to tuberculosis than cattle or goats [31]. In goats, susceptibility may vary according to host factors and mycobacterialecotypes, including *Mycobacterium caprae*, which has been reported mainly in Europe [32]. The prevalenceestimated in our study is comparable to previous slaughterhouse-based Algerian reports by Ayad et al. [15] and Dergal et al. [18], but lower than values reported in Algerian studies using culture or PCR confirmation [16,33,34]. This difference probably reflects methodological heterogeneity, because abattoir inspection detects visible lesions, whereas laboratory methods can identify infection more specifically. International comparisons also show wide variability, with higher prevalence reported in Egypt [35,36], Iraq [37], Tunisia [38], and Iran [39]. Such variation may result from differences in livestock production systems, animal movement, breed, age, immunestatus, surveillance intensity, and diagnostic methods.

It is well established that *Mycobacterium tuberculosis* is the primary causative agent of human TB, whereas *M. bovis* accounts for a smaller but epidemiologically important fraction of zoonotic TB. The observed seasonal pattern in bTB-like lesions may be associated with livestock management practices, particularly during autumn and winter when animals are commonly confined within enclosures. Such conditions increase animal density and the frequency of close contact, thereby facilitating the transmission. In the present study, the average number of human TB cases was approximately 220 per year, which is higher than the 181 cases reported in the previous study conducted between 2017 and 2019 [16]. In another investigation, Tazerart et al. [40] identified 4 out of 98 MTBC isolates collected in two regions of Algeria between 2015 and 2018, which were confirmed as *M. bovis* by whole-genome sequencing. Several studies have also reported relatively high proportions of zoonotic tuberculosis, ranging from 8% in Iraq [41] and 5.36% in Egypt [42] to 3.3% in Lebanon [43], particularly among at-risk populations such as farmers and slaughterhouse workers. The positive correlation observed here between bovine pulmonary tuberculosis-like lesions and human EPTB supports the hypothesis of shared environmental or occupational exposure, but it cannot establish direct zoonotic transmission without individual-level microbiological and molecular linkage. Potential transmission routes include inadequate hand hygiene or disinfection among dairy-farm workers after handling livestock potentially infected with *M. bovis* [36], and consumption of contaminated raw or unpasteurized milk [44]. In the current investigation, the strong positive correlation (r = 0.81) between bovine respiratory lesions due to TB and human EPTB supports the hypothesis of a shared pathogen environment, likely driven by close human–animal contact and the consumption of raw dairy products. This highlights the need for further research to clarify how these factors influence public health. In Algeria, the sale of raw milk remains common in rural and mountainous areas, where it is widely used for the production of traditional dairy products, thereby increasing the risk of infection from contaminated milk. It appears that the available statistics on TB in ruminants may underestimate the true disease burden. Despite the implementation of several surveillance and control programs in Algeria, including IDT testing, the culling of positive animals, and routine slaughterhouse inspections, animal tuberculosis continues to represent a persistent public-health concern. According to the World Organization for Animal Health, a country is designated as officially free from tuberculosis when the percentage of cattle herds confirmed as infected remains below 0.1% per year for six consecutive years [45]. Therefore, close communication and collaboration between animal- and public-health authorities remain essential for risk assessment and identification of effective preventive and control measures.

Seasonal patterns should also be interpreted in light of husbandry and climate. In the current study, cattle showed higher carcass and lung lesion occurrence during autumn, which may coincide with the onset of more confined management and increased animal contact. Similar seasonal variation has been reported in Algeria and other settings [46,47]. Climatic factors may influence bTB epidemiology by affecting animal housing, herd aggregation, and the environmental persistence of mycobacterial [48,49]. The study areas are located in a region characterized by a humid, temperate Mediterranean climate, with average temperatures ranging from approximately 10 °C to 13 °C between November and February. Such conditions may favor prolonged survival of mycobacteria in the environment and facilitate indirect transmission, although this interpretation remains ecological and requires confirmation through farm-level studies [50].

In the context of zoonoses, estimating economic losses is essential for guiding the efficient allocation of public funds and prioritizing disease-control programs. Several studies have examined the economic impact of bovine tuberculosis by estimating losses incurred at farm and slaughterhouse levels [12,51]. Bovine tuberculosis, mainly associated with *Mycobacterium bovis*, can reduce livestock productivity through lower meat and milk output, increased culling, and condemnation of affected organs or carcasses [52,53]. In addition to its economic impact, the disease remains relevant for public health because of possible zoonotic transmission through occupational exposure or consumption of contaminated animal products.

All tuberculosis-like lesions observed in carcasses, lungs, and livers were deemed unfit for human consumption and led to partial or total condemnation according tostandard slaughterhouse procedures. The lack of previous Algerian studies assessing the economic losses caused by tuberculosis-like lesions in ruminants makes it difficult to fully evaluate the national burden of this zoonotic disease on the livestock sector. The retrospective survey, conducted over a six-year period in two provinces of northern Algeria and involving three ruminant species, helps explain the considerable economic impact observed. According to our findings, the estimated total economic losses attributed to tuberculosis-like lesions in slaughterhouses over this six-year period exceeded €14.16 million. The total cost of losses due to tuberculosis in ruminants, both at the public and farm levels, isconsiderable, amounting to USD 403,117 in Turkey (case of Samsun province) [54]. Similarly, losses resulting from the condemnation of tuberculosis-infected cattle carcasses in slaughterhouses in the municipality of Pirassununga (São Paulo, Brazil) have been estimated at approximately €30,148 [55]. Similar observations were reported by Elmonir and Ramadan [56], who found that the direct economic losses associated with the condemnation of meat and liver affected by tuberculosis lesions in slaughtered cattle and buffaloes in the Mid-Delta region of Egypt amounted to USD 28,544.30. Likewise, Atawalna et al. [57] reported that the total direct economic loss due to carcasses and organs condemnation in ruminants slaughtered at the Bolgatanga Municipal Abattoir in Ghana was lower (USD 18,693.06) than that estimated in our study. In addition, Ejeh et al. [58] reported that the economic losses resulting from the condemnation of organs affected by bovine tuberculosis in slaughterhouses in Makurdi, Nigeria, amounted to approximately USD 18,200 during the period from 2008 to 2012. In our study, indirect economic losses accounted for approximately 77% of the total, aligning closely with the results reported by Pérez-Morote et al. [51]. 

According to Pérez-Morote et al. [51], loss of profit, including reduced carcass weight, replacement expenditures, and herd immobilization, represents a major component of the total economic impact on farms and may exceed the direct value of condemned organs. Our results similarly indicate that slaughterhouse seizure data capture only part of the overall economic burden. Differences in reported economic losses between countries may be explained by variation in bovine tuberculosis prevalence, seizure type, market prices, religious or social periods affecting meat demand, compensation systems, and period costs. The variations in the amount of financial losses could be attributed to food inflation in Algeria, which has led to higher prices for food and meat products, as well as increased production and logistics costs, such as transport and animal feed. In Algeria, edible organs such as lungs and livers are highly valued by consumers, so condemnation directly affects butcher and farmer income. Althoughour analysis was limited to two provinces, the magnitude of the estimated losses supports the need for wider national assessment and strengthened livestock tuberculosis control measures.

Our findings indicate that bTB-compatible lesions continue to exert a substantial economic burden on the livestock sector in northern Algeria. According to the projection model, cumulative losses are expected to reach €16.7 million between 2025 and 2030 if surveillance conditions, market prices, and control efforts remain broadly comparable to the retrospective period. The positive slope observed for cattle (b = 0.152) is consistent with the hypothesis ofa gradually increasing economic burden, but the projection should be interpreted cautiously because it is based on a short historical time series. Silva et al. [12] similarly reported substantial projected losses due to carcass condemnation, mainly associated with brucellosis and tuberculosis in bovine and buffalo populations in Brazil. As one of the first investigations to address this topic in Algeria, the present study provides useful baseline evidence, although the limited availability of directly comparable national data constrains cross-study interpretation.

This study has several strengths and limitations. Its strengths include the large number of slaughtered ruminants examined, the six-year retrospective period, the inclusion of two provinces, and the integration of slaughterhouse inspection, IDT results, economic estimates, and human tuberculosis records within a One Health framework. However, the retrospective design relies on routinely collected administrative data, which may contain reporting inconsistencies and incomplete individual-level information. In addition, post-mortem inspection detects tuberculosis-like lesions but does not systematically confirm *M. bovis* infection by culture, PCR, or spoligotyping. Some lesions may therefore have been caused by other members of the MTBC, non-tuberculous mycobacteria, or other granulomatous conditions, leading to possible misclassification or over-estimation of confirmed bovine tuberculosis. The economic projections should also be interpreted cautiously because they depend on assumptions regarding future slaughter volumes, disease prevalence, meat prices, exchange rates, and control-program intensity. Finally, the correlation between animal lesions and human EPTB is ecological and exploratory and does not establishdirectindividual-levelzoonotictransmission.

## 5. Conclusions

This retrospective survey provides one of the first integrated assessments of ruminant tuberculosis-compatible lesions and their economic consequences in slaughterhouses from Bejaia and Jijel, northern Algeria, over the 2019–2024 period. The findings show that cattle represent the main species affected and account for almost the entire estimated financial burden, whereas sheep and goats contribute only marginally. The predominance of pulmonary lesions, the persistence of positive IDT results, and the ecological association with human EPTB support the need for reinforced surveillance under a One Health framework. However, because slaughterhouse inspection identifies tuberculosis-like lesions rather than systematically confirmed *M. bovis* infection, future studies should include bacteriological and molecular confirmation to refine epidemiological attribution. From an economic perspective, the estimated losses indicate that bovine tuberculosis remains not only a veterinary and public-health issue but also a structural constraint for livestock productivity. Strengthening farm-level screening, improving traceability, harmonizing slaughterhouse reporting, and expanding national-scale economic evaluations should be considered priorities for tuberculosis control in Algeria.

## Figures and Tables

**Table 1:** Slaughter statistics andprevalence of tuberculosis-like lesions among ruminants between 2019 and 2024 in Bejaia and Jijel Provinces.

Species Slaughtered	Cattle	Sheep	Goats	All
Slaughtered number	179,549	75,634	112,543	367,726
Slaughtered percentage (%)	48.8	20.6	30.6	100
Number with tuberculosis-like lesions				
Carcass	289	1	1	291
Lung	3120	3	2	3125
Liver	707	4	1	712
Overall prevalence, %(95%CI)				
Carcass	0.16 ^a^ (0.14–0.18)	0.001 ^b^ (0.00–0.01)	0.001 ^b^ (0.00–0.01)	0.08 (0.07–0.09)
Lung	1.74 ^a^ (1.68–1.80)	0.004 ^b^ (0.00–0.01)	0.002 ^b^ (0.00–0.01)	0.85 (0.82–0.88)
Liver	0.39 ^a^ (0.37–0.42)	0.005 ^b^ (0.00–0.01)	0.001 ^b^ (0.18–0.21)	0.19 (0.18–0.21)
Desired absolute precision (d, 5%)				
Carcass	0.0015	0.00025	0.00018	0.0006
Lung	0.0045	0.00048	0.00026	0.0003
Liver	0.0018	0.0005	0.00018	0.0008
IDT number *	24,177	**/**	**/**	24,177
Number with positive IDT	278	/	/	278
Overall prevalence (%)	1.15	/	/	1.15
Desired absolute precision (d, 5%)	0.0015	/	/	0.0015

^a,b^ Values with different superscripts between species are significantly different (*p* ≤ 0.001). CI: 95% Confidenence interval. * Intradermal tuberculin (IDT) test is applied only to dairy cattle.

**Table 2:** Data on cases of human tuberculosis recorded between 2019 and 2024 in the province of Jijel.

Variables	Number of Cases	%	95% CI
EPTB classification			
Lymph node	713	53.9	51.2–56.6
Pleural	100	7.6	6.3–9.1
Peritoneal	156	11.8	10.2–13.7
Others *	353	26.7	24.4–29.2
Patient age ^1^			
15–24	203	31.5	28.1–35.2
25–45	286	44.4	40.6–48.3
>46	155	24.1	20.9–27.5
Gender ^1^		39.0	35.2–42.8
Male	247	39.0	35.2–42.8
Female	387	61.0	57.2–64.8
Year ^1^			
2019	240	18.2	16.2–20.3
2020	226	17.1	15.2–19.2
2021	223	16.9	14.9–19.0
2022	239	18.1	16.1–20.2
2023	197	14.9	13.1–16.9
2024	197	14.9	13.1–16.9

* Primary infection, osteoarticular, urogenital, meningeal, etc. ^1^ The data relate to lymph node, pleural, and peritoneal tuberculosis.Values are observed cases extracted from medical records. Percentages were calculated within each block; Wilson 95% confidence intervals are reported descriptively and do not imply causal inference. *p*-values are not applicable for descriptive breakdowns without denominators/time-at-risk.

**Table 3:** Multivariate logistic regression analysis of tuberculosis-like lesion occurrence and associated risk factors among ruminants in Bejaia and Jijel provinces.

Variable	Odds Ratio	CI (95%)	*p*-Value	Interpretation
Species				
Cattle	1.00 (Ref.)	-	-	Most affected species
Sheep	0.03	<0.01–0.06	<0.001	Negligible risk (Spillover)
Goats	0.02	<0.01–0.05	<0.001	Negligible risk (Spillover)
Season				
Winter	1.00 (Ref.)	-	-	High-risk season
Spring	0.95	0.76–1.18	0.583	Stable *vs*. Winter
Summer	0.82	0.63–1.06	0.126	Reduced risk (Outdoor/UV)
Autumn	1.18	0.92-1.5	0.058	High risk (Stabilizing onset)
Years				
2019	0.71	0.58–0.87	<0.001	Lower initial risk
2020	0.89	0.72–1.1	0.264	Moderate (COVID-19 impact)
2021	1.04	0.85–1.26	0.673	High plateau
2022	0.98	0.8–1.2	0.892	Stable
2023	0.85	0.68–1.06	0.189	Moderate decrease
2024	1.00 (Ref.)	-	-	Recent peak
Study area				
Bejaia	1.00 (Ref.)	-	-	Hyper-endemic area
Jijel	0.68	0.52–0.89	0.009	Significantly lower risk
Meteorological variables				
Temperature (+1 °C)	0.96	0.91–1.01	0.073	Protective trend (heat)
Rainfall (+10 mm)	1.06	0.98–1.14	0.023	Aggravating factor
Relative humidity	1.02	0.98–1.07	0.334	Effect masked by rainfall

OR: odds ratio; CI: confidence interval; Ref.: reference method.

**Table 4:** Total condemned organs and carcasses (kilograms) due to tuberculosis-like lesions (2019–2024) and theircorresponding direct and indirect economic losses (euros) in slaughterhouses of Bejaia and Jijel provinces.

Estimation	Cattle	Sheep	Goats	All
Organ condemnation with tuberculosis-like lesions (kg)				
Lung	10,366	5	3	10,374
Liver	4056	5	0	4061
Direct economic losses due to organ condemnation caused by tuberculosis-like lesions (Euro)	2,627,081	4185	858	2,632,124
Carcass condemnation with tuberculosis-like lesions (kg)	31,739	16	0	31,755
Direct economic losses due to carcass condemnation caused by tuberculosis-like lesions (euros)	601,512	333	0	601,845
Indirect economic losses due to tuberculosis-like lesions of ruminant (euros)	10,910,095	10,886	3269	10,924,250
Total economic losses (euros)	14,138,688	15404	4127	14,158,219

Direct economic losses due to organ condemnation caused by tuberculosis-like lesions: DELo = (MAS × PLr × CLr) + (MAS × PLu × CLu). Where MAS = mean annual cattle slaughtered at the study area; PLr = percentage of liver condemned; PLu = percentage of lung condemned; CLr = mean cost of a liver; and CLu = mean cost of a lung. Direct economic losses due to carcass condemnation caused by tuberculosis-like lesions: DELc = NC × AWC × ACP. Where DEL = direct economic losses due to carcass condemnation; NC = number of condemned carcasses; AWC = average weight of carcasses (kg); and ACP = average carcass price (Euro/kg). Indirect economic losses due to liver tuberculosis-like lesions in ruminant: IEL = MAS × (ACW × 10%) × MCM × P. Where IEL = indirect economic losses; MAS = mean annual number of ruminants slaughtered in the study area; ACW = average carcass weight; PCW = percentage of carcass weight reduction (ACW × 10%); MCM = mean cost of 1 kg of meat in the study area (euros); and P = prevalence of tuberculosis-like lesions in the study area. Total economic losses: TEC = DELo + DELc + IEL.

**Table 5:** Projection of economic losses (euros) due to ruminant tuberculosis between 2025 and 2030 in slaughterhouses of Bejaia and Jijel provinces.

Years	Projection of Economic Losses due to Ruminant Tuberculosis (Euros)			
	Cattle	Sheep	Goats	All
2025	2,659,697	2898	776	2,663,371
2026	2,714,186	2957	792	2,717,935
2027	2,763,175	3010	807	2,766,992
2028	2,807,745	3059	820	2,811,624
2029	2,848,683	3104	832	2,852,618
2030	2,886,578	3145	843	2,890,565
Total	16,680,064	18,173	4869	16,703,106

## Data Availability

Data is contained within the article.

## Abbreviations

The following abbreviations are used in this manuscript:

TB	Tuberculosis
MBTC	Mycobacterium tuberculosis complex
IDT	Intradermal tuberculin
N	Required sample size
P	Prevalence
D	Desired absolute precision
DZD	Algerian Dinar
MAS	Mean annual cattle slaughtered in thestudy area
PLr	Percentage of livers condemned
PLu	Percentage of lungs condemned
CLr	Mean cost of a liver
CLu	Mean cost of a lung
DEL	Direct economic loss due to carcass condemnation
NC	Number of condemned carcasses
ACW	Average carcass weight
ACP	Average carcasses price
IEL	Indirect economic losses
MAS	Mean annual ruminants slaughtered in thestudy area
PCW	Percentage of carcass weight reduction
MCM	Mean cost of 1 kg of meat
Loss*_t_*	Total annual economic loss

## References

[1] Kardjadj M., Luka P.D. (2016). Current situation of milk and red meat industry in Algeria. J. Nutr. Food Sci..

[2] Wilson C. (2020). Animal tuberculosis. Clinical Tuberculosis.

[3] Da Silva Cezar R.D., Lucena-Silva N., Filho A.F.B.B., de Melo Borges J., de Oliveira P.R.F., Lúcio É.C., Arruda-Lima M., de Assis Santana V.L., Junior J.W.P. (2016). Molecular detection of *Mycobacterium bovis* in cattle herds of Pernambuco State, Brazil. BMC Vet. Res..

[4] Thapa J., Paudel S., Sadaula A., Shah Y., Maharjan B., Kaufman G. (2016). *Mycobacterium orygis*-associated tuberculosis in free-ranging rhinoceros, Nepal, 2015. Emerg. Infect. Dis..

[5] Ullah A., Hafeez F., Taj R., Gul S., Khan I., Faheem B., Ahmad M., Ullah R., Ahmad A., Hanif M. (2017). Comparative study on detection of *Mycobacterium bovis* infection in bovine tuberculous lesions. Sarhad J. Agric..

[6] Szacawa E., Radulski Ł., Weiner M., Szulowski K., Krajewska-Wędzina M. (2025). *Mycobacterium tuberculosis* complex infections in animals: A comprehensive review of species distribution and laboratory diagnostic methods. Pathogens.

[7] Carneiro P.A.M., Pasquatti T.N., Lima D.A.R., Rodrigues R.A., Takatani H., Silva C.B.D.G., Kaneene J.B. (2022). Milk contamination by *Mycobacterium tuberculosis* complex and implications for public health in Amazonas, Brazil. J. Food Prot..

[8] Sawyer J., Rhodes S., Jones G.J., Hogarth P.J., Vordermeier H.M. (2023). *Mycobacterium bovis* and its impact on human and animal tuberculosis. J. Med. Microbiol..

[9] Olea-Popelka F.J., Muwonge A., Perera A., Dean A.S., Mumford E., Erlacher-Vindel E., Cosivi O. (2017). Zoonotic tuberculosis in human beings caused by *Mycobacterium bovis*: A call for action. Lancet Infect. Dis..

[10] Vayr F., Martin-Blondel G., Savall F., Soulat J.M., Deffontaines G., Herin F. (2018). Occupational exposure to *Mycobacterium bovis*: A systematic review. PLoS Negl. Trop. Dis..

[11] Hotzel M.J., Vandresen B. (2022). Brazilians’ attitudes to meat consumption and production: Present and future challenges to the sustainability of the meat industry. Meat Sci..

[12] Silva W.C.D., Camargo R.N.C., Silva É.B.R., Silva J.A.R., Picanço M.L.R., Santos M.R.P., Lourenço J.D.B. (2023). Economic losses due to condemnation of cattle and buffalo carcasses in northern Brazil. PLoS ONE.

[13] Clemmons E.A., Alfson K.J., Dutton J.W. (2021). Transboundary animal diseases: An overview of 17 diseases with potential for global spread and serious consequences. Animals.

[14] Ukwaja K.N., Alobu I., Igwenyi C., Hopewell P.C. (2013). Patient and household costs associated with tuberculosis care in Nigeria. PLoS ONE.

[15] Ayad A., Bensid A., Benabdelhak A.C., Ait-Yahia F., Dergal N.B. (2020). First report on tuberculosis based on slaughterhouse data in Bejaia Province, Algeria: A 10-year retrospective survey. Kocatepe Vet. J..

[16] Damene H., Tahir D., Diels M., Berber A., Sahraoui N., Rigouts L. (2020). Broad diversity of *Mycobacterium tuberculosis* complex strains isolated from humans and cattle in Northern Algeria suggests a zoonotic transmission cycle. PLoS Negl. Trop. Dis..

[17] Djafar Z.R., Benazi N., Bounab S., Sayhi M., Diouani M.F., Benia F. (2020). Distribution of seroprevalence and risk factors for bovine tuberculosis in East Algeria. Prev. Vet. Med..

[18] Dergal N.B., Ghermi M., Imre K., Morar A., Acaroz U., Arslan-Acaroz D., Ayad A. (2023). Estimated prevalence of tuberculosis in ruminants from slaughterhouses in Constantine Province, Northeastern Algeria. Life.

[19] Bensid A. (2018). Hygiène et Inspection des Viandes Rouges.

[20] Thrusfield M., Christley R. (2018). Veterinary Epidemiology.

[21] Ogunrinade A., Ogunrinade B.I. (1980). Economic importance of bovine fascioliasis in Nigeria. Trop. Anim. Health Prod..

[22] Kwaghe A.V., Ameh A.J., Ambali A.G., Kudi A.C., Kachalla M.G. (2015). Prevalence and economic losses from bovine tuberculosis in Maiduguri, Nigeria. Int. J. Life Sci..

[23] Hyndman R.J., Athanasopoulos G. (2018). Forecasting: Principles and Practice.

[24] Goodwin T.M., Quiroz R., Kohn R. (2024). Dynamic linear regression models for forecasting time series with semi-long memory errors. arXiv.

[25] Thoen C.O., LoBue P.A., Enarson D.A., Kaneene J.B., de Kantor I.N. (2009). Tuberculosis: A re-emerging disease in animals and humans. Vet. Ital..

[26] Lombard J.E., Patton E.A., Gibbons-Burgener S.N., Klos R.F., Tans-Kersten J.L., Carlson B.W., Robbe-Austerman S. (2021). Human-to-cattle transmission of *Mycobacterium tuberculosis* complex in the United States. Front. Vet. Sci..

[27] Chakaya J., Khan M., Ntoumi F., Aklillu E., Fatima R., Mwaba P., Zumla A. (2021). Global Tuberculosis Report2020: Reflections on the global TB burden, treatment and prevention efforts. Int. J. Infect. Dis..

[28] Mimoune N., Hamiroune M., Boukhechem S., Mecherouk C., Harhoura K., Khelef D., Kaidi R. (2022). Pathological findings in cattle slaughtered in Northeastern Algeria and associated risk factors. Vet. Sci..

[29] Belakehal F., Moser I., Naim M., Zenia S., Hamdi T.M. (2021). Tuberculosis lesions of bovine carcasses in Algerian municipal abattoirs and associated risk factors. J. Anim. Health Prod..

[30] Muñoz-Mendoza M., Romero B., Del Cerro A., Gortázar C., García-Marín J.F., Menéndez S., Balseiro A. (2016). Sheep as a potential source of bovine tuberculosis TB: Epidemiology, pathology and evaluation of diagnostic techniques. Transbound. Emerg. Dis..

[31] Caswell J.L., Williams K.J., Jubb K.V.F., Kennedy P.C., Palmer N.C. (2007). Respiratory system. Pathology of Domestic Animals.

[32] Vidal E., Grasa M., Perálvarez T., Martín M., Mercader I., de Val B.P. (2018). Transmission of tuberculosis caused by *Mycobacterium caprae* between dairy sheep and goats. Small Rumin. Res..

[33] Tazerart F., Saad J., Sahraoui N., Yala D., Niar A., Drancourt M. (2021). Whole genome sequence analysis of *Mycobacterium bovis* cattle isolates from Algeria. Pathogens.

[34] Belakehal F., Barth S.A., Menge C., Mossadak H.T., Malek N., Moser I. (2022). Evaluation of the discriminatory power of spoligotyping and 19-locus mycobacterial interspersed repetitive unit-variable number tandem repeat analysis (MIRU-VNTR) of *Mycobacterium bovis* strains isolated from cattle in Algeria. PLoS ONE.

[35] Elnaker Y.F., ElSharawy N.T., Daib M.S., ElGedawy A.A., Ibrahim N.A. (2018). Conventional and molecular detection of *Mycobacterium bovis* in Aburden angus cattle and human contact in the new valley governorate, Egypt. Assiut Vet. Med. J..

[36] Lobna M.A.S., Nasr E.A. (2015). Conventional and molecular detection of *Mycobacterium bovis* in cow milk and its public health hazard. Int. J. Adv. Res..

[37] Aliraqi O.M.M., Al-Jammaly M., Al-Hankawi O., Al-Farwachi M.I., Dahl M.O. (2020). Preliminary prevalence and risk factors of *Mycobacterium bovis* in local and imported breeds of cattle and buffaloes in Mosul City, Iraq. Egypt. J. Vet. Sci..

[38] Lamine-Khemiri H., Martínez R., García-Jiménez W.L., Benítez-Medina J.M., Cortés M., Hurtado I., Abassi M.S., Khazri I., Benzarti M., Hermoso-de-Mendoza J. (2014). Genotypic characterization by spoligotyping and VNTR typing of Mycobacterium bovis and Mycobacterium caprae isolates from cattle of Tunisia. Trop. Anim. Health Prod..

[39] Mosavari N., Feizabadi M.M., Jamshidian M., Shahpouri M.R.S., Forbes K.J., Pajoohi R.A., Keshavarz R., Taheri M.M., Tadayon K. (2011). Molecular genotyping and epidemiology of *Mycobacterium bovis* strains from cattle in Iran. Vet. Microbiol..

[40] Tazerart F., Saad J., Niar A., Sahraoui N., Drancourt M. (2021). *Mycobacterium bovis* pulmonary tuberculosis, Algeria. Emerg. Infect. Dis..

[41] Barak S. (2012). Incidence of bovine tuberculosis and its public health hazards in a dairy cattle station in Iraq. Al-Anbar J. Vet. Sci..

[42] Abdelsadek H.A., Sobhy H.M., Mohamed K.F., Hekal S.H.A., Dapgh A.N., Hakim A.S. (2020). Multidrug-resistant strains of Mycobacterium complex species in Egyptian farm animals, veterinarians, and farm and abattoir workers. Vet. World.

[43] Bedrossian N., Hamze M., Rahmo A.K., Jurjus A., Saliba J., Dabboussi F., Karam W. (2013). *Mycobacterium tuberculosis* spoligotypes circulating in the Lebanese population: A retrospective study. East. Mediterr. Health J..

[44] Çavuşoğlu C., Yılmaz F.F. (2017). Molecular epidemiology of human *Mycobacterium bovis* infection in Aegean Region, Turkey. Mikrobiyol. Bul..

[45] Gordejo F.R., Vermeersch J.P. (2006). Towards eradication of bovine tuberculosis in the European Union. Vet. Microbiol..

[46] Hadef A., Righi S., Boucheikhchoukh M., Bouzid C.E. (2022). Pattern and major reasons of cattle red offal condemnation in the slaughterhouse of the arid region of El Oued, Algeria. Agriculture.

[47] Ejeh E.F., Markus I.F., Ejeh A.S., Musa J.A., Lawan F.A., Ameh J.A., Cadmus S.I.B. (2013). Seasonal prevalence of bovine tuberculous lesions in cattle slaughtered in Yola abattoirs. Bangladesh J. Vet. Med..

[48] Gong Q.L., Chen Y., Tian T., Wen X., Li D., Song Y.H., Zhang X.X. (2021). Prevalence of bovine tuberculosis in dairy cattle in China during 2010–2019: A systematic review and meta-analysis. PLoS Negl. Trop. Dis..

[49] Rhodes S.G., De Leij F.A., Dale J.W. (2007). Protozoa as an environmental reservoir of bovine tuberculosis. Trends Microbiol..

[50] Ghebremariam M.K., Michel A.L., Vernooij J.C.M., Nielen M., Rutten V. (2018). Prevalence of bovine tuberculosis in cattle, goats and camels of traditional livestock-raising communities in Eritrea. BMC Vet. Res..

[51] Pérez-Morote R., Pontones-Rosa C., Gortázar-Schmidt C., Muñoz-Cardona Á.I. (2020). Economic impact of bovine tuberculosis on livestock farms in South-Western Spain. Animals.

[52] Brahma D., Narang D., Chandra M., Filia G., Singh A., Singh S.T. (2019). Diagnosis of bovine tuberculosis by comparative intradermal tuberculin test, interferon gamma assay and esxB PCR. Open J. Vet. Med..

[53] Kemal J., Sibhat B., Abraham A., Terefe Y., Tulu K.T., Welay K., Getahun N. (2019). Bovine tuberculosis in eastern Ethiopia: Prevalence, risk factors and public health importance. BMC Infect. Dis..

[54] Şentürk B., Akçay A., Sarıözkan S. (2020). Cost of bovine tuberculosis at public and farm levels in Samsun Province, Turkey. Vet. J. Mehmet Akif Ersoy Univ..

[55] Homem V.S.F., Morais Higa Z.M., Neto J.S.F. (2016). Proposed model to study the economic impact of bovine brucellosis and tuberculosis: A case study in Brazil. Semin. Cienc. Agrar..

[56] Elmonir W., Ramadan H. (2016). Abattoir-based prevalence, economic losses and veterinarians’ high-risk practices of bovine tuberculosis in Mid-Delta of Egypt. Alex. J. Vet. Sci..

[57] Atawalna J., Gbordzi M., Emikpe B.O., Anyorigeyah T. (2016). Whole carcass and organ condemnation and associated financial losses in ruminants slaughtered at the Bolgatanga municipal abattoir, Ghana. Int. J. Vet. Sci..

[58] Ejeh E.F., Raji M.A., Bello M., Lawan F.A., Francis M.I., Kudi A.C., Cadmus S.I.B. (2014). Prevalence and direct economic losses from bovine tuberculosis in Makurdi, Nigeria. Vet. Med. Int..

